# Exome Sequencing Reveals Novel Variants and Expands the Genetic Landscape for Congenital Microcephaly

**DOI:** 10.3390/genes12122014

**Published:** 2021-12-18

**Authors:** Mateusz Dawidziuk, Tomasz Gambin, Ewelina Bukowska-Olech, Dorota Antczak-Marach, Magdalena Badura-Stronka, Piotr Buda, Edyta Budzynska, Jennifer Castaneda, Tatiana Chilarska, Elzbieta Czyzyk, Anna Eckersdorf-Mastalerz, Jolanta Fijak-Moskal, Dorota Gieruszczak-Bialek, Ewelina Glodek-Brzozowska, Alicja Goszczanska-Ciuchta, Malgorzata Grzeszykowska-Podymniak, Barbara Gurda, Anna Jakubiuk-Tomaszuk, Ewa Jamroz, Magdalena Janeczko, Dominika Jedlińska-Pijanowska, Marta Jurek, Dagmara Karolewska, Adela Kazmierczak, Teresa Kleist, Iwona Kochanowska, Malgorzata Krajewska-Walasek, Katarzyna Kufel, Anna Kutkowska-Kaźmierczak, Agata Lipiec, Dorota Maksym-Gasiorek, Anna Materna-Kiryluk, Hanna Mazurkiewicz, Michał Milewski, Tatsiana Pavina-Guglas, Aleksandra Pietrzyk, Renata Posmyk, Antoni Pyrkosz, Mariola Rudzka-Dybala, Ryszard Slezak, Marzena Wisniewska, Zofia Zalewska-Miszkurka, Elzbieta Szczepanik, Ewa Obersztyn, Monika Bekiesinska-Figatowska, Pawel Gawlinski, Wojciech Wiszniewski

**Affiliations:** 1 Department of Medical Genetics, Institute of Mother and Child, 01-211 Warsaw, Poland; mateusz.dawidziuk@imid.med.pl (M.D.); tomasz.gambin@imid.med.pl (T.G.); jennifer.castaneda@imid.med.pl (J.C.); marta.jurek@imid.med.pl (M.J.); anna.kutkowska@imid.med.pl (A.K.-K.); michal.milewski@imid.med.pl (M.M.); ewa.obersztyn@imid.med.pl (E.O.); pawel.gawlinski@imid.med.pl (P.G.); 2 Department of Medical Genetics, Poznan University of Medical Sciences, 60-806 Poznan, Poland; ewe.bukowska@gmail.com (E.B.-O.); bstronka@gmail.com (M.B.-S.); akiryluk@ump.edu.pl (A.M.-K.); m.wisniewska@genesis.pl (M.W.); 3 Clinic of Paediatric Neurology, Institute of Mother and Child, 01-211 Warsaw, Poland; dorantmar@wp.pl (D.A.-M.); alicja.goszczanska@imid.med.pl (A.G.-C.); agata.lipiec@imid.med.pl (A.L.); hanna.mazurkiewicz@imid.med.pl (H.M.); mariola.rudzka@imid.med.pl (M.R.-D.); zofia.zalewska@imid.med.pl (Z.Z.-M.); elzbieta.szczepanik@imid.med.pl (E.S.); 4 Department of Pediatrics, Nutrition, and Metabolic Diseases, The Children’s Memorial Health Institute, 04-730 Warsaw, Poland; p.buda@ipczd.pl; 5 Department of Clinical Genetics, Central Clinical Hospital, Medical University of Lodz, 92-213 Lodz, Poland; e.budzynska@csk.umed.pl (E.B.); ae.mastalerz@csk.umed.lodz.pl (A.E.-M.); 6 Department of Genetics, Polish Mother’s Memorial Hospital Research Institute, 93-338 Lodz, Poland; tatianagen5@wp.pl; 7 Clinical Department of Child Neurology, Clinical Central Hospital No. 2 in Rzeszow, 35-301 Rzeszow, Poland; el.czyzyk@wp.pl (E.C.); ebrzozowska7@gmail.com (E.G.-B.); 8 Department of Medical Genetics, Jagiellonian University Medical College, 30-663 Krakow, Poland; j.fijak.moskal@gmail.com; 9 Department of Paediatrics, Medical University of Warsaw, 01-184 Warsaw, Poland; dorekgier@gazeta.pl; 10 Department of Nephrology, Kidney Transplantation and Hypertension, The Children’s Memorial Health Institute, 04-730 Warsaw, Poland; m.podymniak-grzeszykowska@ipczd.pl; 11 Department of Developmental Neurology, Poznan University of Medical Sciences, 60-355 Poznan, Poland; bgurda@interia.pl; 12 Department of Pediatric Neurology and Rehabilitation, Medical University of Bialystok, 15-274 Bialystok, Poland; ajaktom@gmail.com; 13 Department of Pediatric and Neurology of Developmental Age, Saint John Paul II Upper Silesian Child Health Centre, The Independent Public Clinical Hospital No. 6 of the Medical University of Silesia, 40-055 Katowice, Poland; jamroz.ewa5@gmail.com; 14 Department of Genetics, Institute of Pediatrics, Jagiellonian University Medical College, 30-663 Krakow, Poland; mlegutko@cm-uj.krakow.pl; 15 Department of Neonatology and Neonatal Intensive Care, The Children’s Memorial Health Institute, 04-730 Warsaw, Poland; d.jedlinska-pijanowska@ipczd.pl; 16 Specialist Mother and Child Hospital Complex in Poznan, 61-825 Poznan, Poland; dagakarolewska@gmail.com; 17 Independent Public Healthcare Center, Department of Pediatric Neurology, Multidisciplinary Hospital, 67-100 Nowa Sol, Poland; adnur@op.pl; 18 Department of Pediatric Neurology Neurological Outpatient Clinic for Children, Municipal Hospital Complex, 41-500 Chorzow, Poland; teresa_kleist@tlen.pl; 19 Individual Medical Practice in Pediatric Neurology, 70-592 Szczecin, Poland; ikochanowska@onet.eu; 20 Department of Medical Genetics, The Children’s Memorial Health Institute, 04-730 Warsaw, Poland; malgorzata.krajewskawalasek@gmail.com; 21 Department of Genetics and Clinical Immunology, National Institute of Tuberculosis and Lung Diseases, 01-138 Warsaw, Poland; 22 Department of Neonatal and Intensive Care, Medical University of Warsaw, 01-184 Warsaw, Poland; kufel.k@gmail.com; 23 Department of Neonatology, Neonatal Pathology and Intensive Care, The Children’s Memorial Health Institute, 04-730 Warsaw, Poland; dmaksym@wp.pl (D.M.-G.); tatiana.pavina@gmail.com (T.P.-G.); 24 Polish Registry of Congenital Malformations, Department of Medical Genetics, Poznan University of Medical Sciences, 60-806 Poznan, Poland; 25 Department of Genetics and Pathomorphology, Faculty of Medicine and Health Sciences, University of Zielona Gora, 65-046 Zielona Gora, Poland; a.pietrzyk@cm.uz.zgora.pl; 26 Department of Clinical Genetics, Medical University of Bialystok, 15-089 Bialystok, Poland; rposmyk@gmail.com; 27 Department of Medical Genetics, University of Rzeszow, 35-959 Rzeszow, Poland; antoni.pyrkosz@gmail.com; 28 Department of Genetics, Wroclaw Medical University, 50-368 Wroclaw, Poland; ryszard.slezak@umed.wroc.pl; 29 Department of Diagnostic Imaging, Institute of Mother and Child, 01-211 Warsaw, Poland; monika.bekiesinska@imid.med.pl

**Keywords:** molecular genetics, neurology, high-throughput nucleotide sequencing, medical genetics, human genetics

## Abstract

Congenital microcephaly causes smaller than average head circumference relative to age, sex and ethnicity and is most usually associated with a variety of neurodevelopmental disorders. The underlying etiology is highly heterogeneous and can be either environmental or genetic. Disruption of any one of multiple biological processes, such as those underlying neurogenesis, cell cycle and division, DNA repair or transcription regulation, can result in microcephaly. This etiological heterogeneity manifests in a clinical variability and presents a major diagnostic and therapeutic challenge, leaving an unacceptably large proportion of over half of microcephaly patients without molecular diagnosis. To elucidate the clinical and genetic landscapes of congenital microcephaly, we sequenced the exomes of 191 clinically diagnosed patients with microcephaly as one of the features. We established a molecular basis for microcephaly in 71 patients (37%), and detected novel variants in five high confidence candidate genes previously unassociated with this condition. We report a large number of patients with mutations in tubulin-related genes in our cohort as well as higher incidence of pathogenic mutations in *MCPH* genes. Our study expands the phenotypic and genetic landscape of microcephaly, facilitating differential clinical diagnoses for disorders associated with most commonly disrupted genes in our cohort.

## 1. Introduction

Microcephaly is caused by reduced brain volume and is defined as the deviation of an individual’s occipitofrontal head circumference (OFC) by more than two standard deviations (SD) below the mean for age, sex and ethnicity. The condition is also commonly associated with neurodevelopmental disorders and frequently associated with neurological features such as intellectual disability (ID) as well as developmental delay (DD) and epilepsy [1]. Microcephaly affects approximately 2% to 3% of the population worldwide [2] with both genetic and environmental etiology. In patients with established etiology of microcephaly, the rate of cases resulting from genetic and environmental causes is close to even at 52% and 48%, respectively [2]. Microcephaly can be present at birth (primary microcephaly—PM) or develop postnatally (secondary microcephaly—SM). This distinction is useful in diagnosis and elucidation of genetic etiology [3]. PM is often a result of defects in neurogenesis, while SM is associated with progressive neurodegenerative disease [4,5]. Microcephaly can present as an isolated clinical symptom but more frequently as a phenotypic feature of a variety of genetic syndromes. As of March 2021 the clinical feature “microcephaly” returns nearly 1600 entries in the Online Mendelian Inheritance in Man (OMIM) database [6]. The genetic and clinical heterogeneity of this condition therefore constitutes a major diagnostic and consequently therapeutic challenge. In order to properly assess the underlying cause of microcephaly, patients require thorough physical examination, analysis of medical records with attention to maternal disease and infections during pregnancy as well as adverse events during labor and diagnostic investigations including brain imaging, biochemical tests and chromosomal and genetic analyses. These steps help discern whether the basis for microcephaly in the patient is fundamentally environmental, genetic or multifactorial, which in turn can help determine further diagnostic and treatment options.

Exome sequencing (ES) has proved highly effective in identifying genetic causes for a range of Mendelian disorders, with a diagnostic rate of up to 50% [7]. The number of genes associated with microcephaly has increased over the last few years, primarily due to broad application of ES for research and diagnostic purposes. For example, the number of known genes causing autosomal recessive primary microcephaly (*MCPH*) has more than doubled from 12 to 28 in 5 years ([8,9], OMIM Phenotypic series: PS251200). Diagnosis using ES does not require prior knowledge of specific genes responsible for clinical phenotype. It is particularly advantageous for accurately and rapidly diagnosing genetically heterogeneous conditions with microcephaly as one of the features that can be caused by mutation in one of several hundred genes. In addition to better understanding the etiology of this specific disorder, identification of novel genes, the mutations of which cause microcephaly, can help elucidate biological mechanisms crucial to brain development and associated diseases.

Three previous studies have used ES to investigate the genetic basis of microcephaly in cohorts of patients presenting with this disorder [10,11,12]. Despite good progress in unraveling the genetic etiology of the condition, a large portion of over 50% of patients presenting with microcephaly remain without specific molecular diagnosis, making the most effective therapeutic measures for the patient difficult to establish. In this study, we carried out ES on 191 Polish patients clinically diagnosed with microcephaly. We established molecular diagnosis where possible and identified 12 previously unassociated potential disease genes. Our work further adds to mounting evidence that ES is the diagnostic method of choice in patients presenting with microcephaly.

## 2. Materials and Methods

We performed ES for a cohort of 191 Polish patients, including 3 sib-pairs, with clinically diagnosed microcephaly. Patients were referred between 2017 and 2019 from different medical centers in Poland and were given comprehensive clinical evaluation in specialized genetic and neurology clinics. Patients enrolled in our project had OFC <−2 SD below average for age and sex and negative history of environmental exposures. All patients underwent brain imaging studies to detect potential brain malformations. Out of 191 patients, 149 of them had either karyotyping or array comparative genomic hybridization analysis (aCGH) performed prior to ES. Informed consent was obtained from participating families, and the study protocol was approved by the Ethics Committee of the Institute of Mother and Child in Warsaw.

Whole-exome sequencing was performed by CeGaT GmbH, Germany. Sequencing libraries were prepared using Agilent SureSelect All Exon V6 sample preparation kits and carried out on Illumina NovaSeq 6000 sequencer, via 2 × 100 bp reads. Genomic data processing was based on an in-house developed pipeline, consisting of read alignment to a hg38 reference genome using Burrows-Wheeler Aligner software (BWA v0.7.16) [13], duplicated reads removal with Picard (v2.11.0) and variant calling with Genome Analysis Toolkit HaplotypeCaller (GATK v4.0b4) [14] through bcbio-nextgen toolkit (v.1.0.5) [15]. Variant annotation was carried out with Ensembl Variant Effect Predictor (v96) [16] using resources from the ClinVar clinical database [17], gnomAD v3.0 population database [18] and dbNSFP v4.1 functional predictions database [19]. Copy number variant (CNV) analysis was carried out with CoNIFER (v0.2.2) [20]. ES quality control was caried out with the Mosdepth (v0.2.4) [21], FastQC (v0.11.5), Bcftools (v1.5) [22], QualiMap (v2.2.2a) [23], Samtools (v1.5) [22] and MultiQC (v1.8) [24].

In this study we used a mixed approach. We first screened probands only, using ES and an in silico microcephaly gene panel to find patients with pathogenic or likely pathogenic variants in known genes associated with the disorder. Then, for selected negative cases both parents underwent ES to discover causative mutations in previously unassociated genes that might lead to microcephaly.

The in silico gene panel used to screen patients for variants in genes associated with microcephaly consisted of over 800 genes. These genes were manually selected from several gene panels customized for patients with microcephaly, and additional genes were selected from various databases such as OMIM or DECIPHER. The detailed gene list with details regarding coverage across all samples is included in Table S2. Selected variants from ES were confirmed by Sanger sequencing using an ABI AB3730 capillary sequencer. Detailed information regarding genes and exon coverage and coverage thresholds for each sample, based on RefSeq genes from NCBI, is available in Table S3. Sequencing quality control data from mentioned tools are available as a MultiQC report in File S4.

Molecular diagnosis for patients was established according to the following classification: (I) definitive diagnosis—the variant found is located in a gene with established association with microcephaly, zygosity and inheritance pattern in the family is in accordance with the underlying disease and the variant is reported in the ClinVar database as a pathogenic or likely pathogenic variant; (II) likely diagnosis—the variant found is located in a gene with established association with microcephaly, zygosity and inheritance pattern in the family is in accordance with the underlying disease and the variant is not reported in the ClinVar database but is predicted to be pathogenic/likely pathogenic by ACMG classification; (III) uncertain diagnosis—the variant is located in a gene with established association with microcephaly, unreported in the ClinVar database (or is reported as a variant of uncertain significance (VUS)) and is predicted to be VUS by the American College of Medical Genetics and Genomics (ACMG) classification; (IV) novel—the variant is located in a gene with no established association with microcephaly, but zygosity and inheritance pattern in the family indicate a possible pathogenic nature and published data suggest that it may interfere with processes relevant to microcephaly etiopathogenesis.

## 3. Results

### 3.1. Clinical Characteristics of the Cohort

We investigated 191 unrelated subjects with microcephaly of suspected genetic etiology, including 97 females and 94 males with average age of 5 years at last clinical evaluation (median: 3.2 years, range: 0 to 18 years). All investigated subjects were determined to have exclusively European descent, with no known consanguinity between parents. PM was suspected in 77 patients (40%) and SM in 110 patients (58%). For the remaining four patients (2%) the time of onset of microcephaly could not be determined. Three subjects (3/191) reported a family history of microcephaly with an affected sibling presenting with similar phenotype and clinically unaffected parents. Many patients presented with additional clinical findings including cognitive impairment (both ID and DD), abnormal muscle tone, epilepsy, dysmorphic facial features and short stature (Table 1). The most frequent findings on brain images were corpus callosum abnormalities, gyrification alterations, delayed myelination and cerebellum abnormalities.

Of 191 total cases, definitive molecular diagnosis of microcephaly was made in 36/191 (19%) cases and a likely diagnosis in a further 35/191 (18%), while in another 11/191 (6%) cases we found variants of unknown significance in genes previously associated with microcephaly. Taken together they give a total of 82/191 cases, or a 43% diagnostic rate. We further found potential novel variants in previously unassociated candidate genes in 11/191 (6%) cases. For the remaining 98/191 (51%) cases we were not able to find causative or potential variants. In 4/191 (2.5%) cases we found more than one likely pathogenic variant in distinct genes linked to microcephaly. Altogether, we identified 132 (of which 115 were unique) variants affecting 65 genes in 93 patients (Table S1). Of the 132 variants, 88 (67%) were missense, 19 (14%) frameshift, 13 (10%) nonsense, 7 (5%) canonical splice site, 4 (3%) located within small nuclear RNA gene and 1 (1%) inframe duplication. Sixty-three variants were of de novo origin, and 72 were not previously reported in the ClinVar database. Following ACMG guidelines [25] 65 of the identified 132 variants (49%) were classified as pathogenic, 29 (22%) as likely pathogenic, another 29 (22%) as VUS and the remaining 9 (7%) variants as likely benign. In total we observed autosomal dominant inheritance in 42/78 (54%) cases, autosomal recessive in 26/78 (33%) cases, X-linked dominant in 7/78 (9%) cases and X-linked recessive in 3/78 (4%) cases. For the remaining 15 cases inheritance was either unknown, as in the case of variants in novel genes (11 cases), or the patient had more than one possible molecular diagnosis (four cases). Detailed information about patients’ phenotype as well as genetic findings are in Table S1. Taken together we found pathogenic/likely pathogenic variants or VUS in 54 different genes associated with microcephaly, as well as novel variants in 12 previously unassociated genes. Results of CNV analysis with Conifer software were examined for occurrence of heterozygous variants in dominant genes as well as homozygous variants in recessive genes linked with microcephaly. In patients with known pathogenic heterozygous variants in genes with recessive inheritance mode, we also looked at Conifer data in search for possible CNVs in the gene of interest; however, we did not find such cases in our cohort.

### 3.2. Definitive and Likely Diagnosis Cases

In our cohort, we found variants in a larger number of genes encoding tubulins previously linked with microcephaly. Two of these genes in particular, *TUBA1A* (five patients) and *TUBB3* (five patients), showed the highest number of pathogenic mutations. Out of 71 patients with definitive or likely diagnosis, 15 (21%) overall had a causative variant in a tubulin gene. Aside from tubulins, other frequently mutated genes in our cohort are *CTNNB1*, *FOXG1* and *TSEN54*, each identified in four out of 71 cases (6%), and *ASPM*, *EFTUD2* and *PDHA1*, each in three out of 71 patients (4%). For conditions associated with the *CTNNB1*, *FOXG1*, *EFTUD2* and *PDHA1* genes, mutations are normally inherited in a dominant manner, and all our patients were found with de novo heterozygous variants. All patients with *TSEN54* mutations, which cause pontocerebellar hypoplasia type 2A (*PCH2A*), an autosomal recessive disorder, shared the same variant NM_207346.3: c.919G > T: p.(Ala307Ser) in homozygous state inherited from carrier parents. This variant is over-represented in the European population (excluding Finnish) with a frequency of 1.7 × 10^−3^, in comparison to 2 × 10^−4^ in other populations, according to gnomAD v3 database. All *PCH2A* patients we report here have progressive microcephaly with average SD at birth nearing 0 and −4.9 at the time of last examination (1 year 9 months average) as well as cerebellum and/or brainstem hypoplasia, DD, hypertonia and epilepsy.

Overall we found pathogenic/likely pathogenic variants in 45 unique genes related to microcephaly, of which 27 have autosomal dominant inheritance, 12 autosomal recessive inheritance, 4 X-linked dominant inheritance and 1 each X-linked recessive and X-linked inheritance. In these genes we identified 54 missense, 16 frameshift, 13 nonsense, 7 canonical splice site and 4 variants located within the small nuclear RNA gene.

### 3.3. Dual Molecular Diagnosis

Among 71/191 cases with definitive or likely diagnosis, we found dual molecular diagnoses in four patients (Table 2).

In patient T50 with SM (−3.1 SD), DD, ID, radiological findings of Dandy–Walker malformation and anterior commissure agenesis as well as dysmorphic facial features, we found a heterozygous de novo variant NM_178012.5: c.1171C>T: p.(Arg391Cys) in *TUBB2B* gene and a hemizygous, maternally inherited variant NM_031407.7: c.11434G>A: p.(Val3812Met) in *HUWE1*. Neither are reported in the ClinVar database. The former mutation causes amino acid substitution and has high deleterious scores in 20/20 prediction algorithms, as well as pathogenic ACMG classification. In addition, a neighboring variant causing the amino acid change p.Arg391His is classified as likely pathogenic in the ClinVar database. The latter mutation was previously classified as VUS under ACMG guidelines. In patient S177 presenting with SM (−3.2 SD), DD, hypotonia and dysmorphic facial features, we identified a heterozygous de novo variant NM_031407.7: c.9208C>T: p.(Arg3070Cys) in *HUWE1* and another de novo heterozygous variant NM_015265.4: c.490G>A: p.(Asp164Asn) in the *SATB2* gene. While the *HUWE1* variant is reported in the ClinVar database as pathogenic/likely pathogenic, the *SATB2* variant was not previously reported though is likely pathogenic according to ACMG classification. In patient S188 with PM (−3.9 SD), bilateral polymicrogyria, DD, epilepsy, spastic tetraplegia, nystagmus, convergent strabismus and cryptorchidism we found two heterozygous de novo variants in two genes encoding ion channel subunits: KCNT1-NM_020822.3: c.1720G>A: p.(Glu574Lys) and GRIN1-NM_007327.4: c.1665G>T: p.(Met555Ile). Both variants have pathogenic classification according to ACMG and are not reported in the ClinVar database. Patient S78 presented clinically with PM (−3.7 SD), DD, epileptic encephalopathy, axial hypotonia, limb hypertonia and EEG abnormalities and has a homozygous mutation NM_024596.5: c.664T>C: p.(Cys222Arg) in the *MCPH1* gene inherited from carrier parents and a heterozygous de novo variant NM_014191.4: c.5630A>G: p.(Asn1877Ser) in the *SCN8A* gene. Both variants are reported in the ClinVar database with conflicting interpretations of pathogenicity, where besides submissions classifying both variants as VUS, the MPCH1 variant has one additional likely benign submission, while *SCN8A* has four pathogenic and two likely pathogenic submissions. Further, under the ACMG guidelines the MPCH1 variant is classified as likely benign, while *SCN8A* is classified as pathogenic.

### 3.4. Novel Gene Associations

We identified variants in 12 genes not previously associated with human diseases. To assess possible status as novel candidate genes, mutations of which may cause microcephaly in our patients, we grouped the genes into two categories, high or low confidence, based on the level of evidence. Results of analysis of the high confidence candidates are presented below; Table S1 shows information on the low confidence candidates.

**Figure 1:**
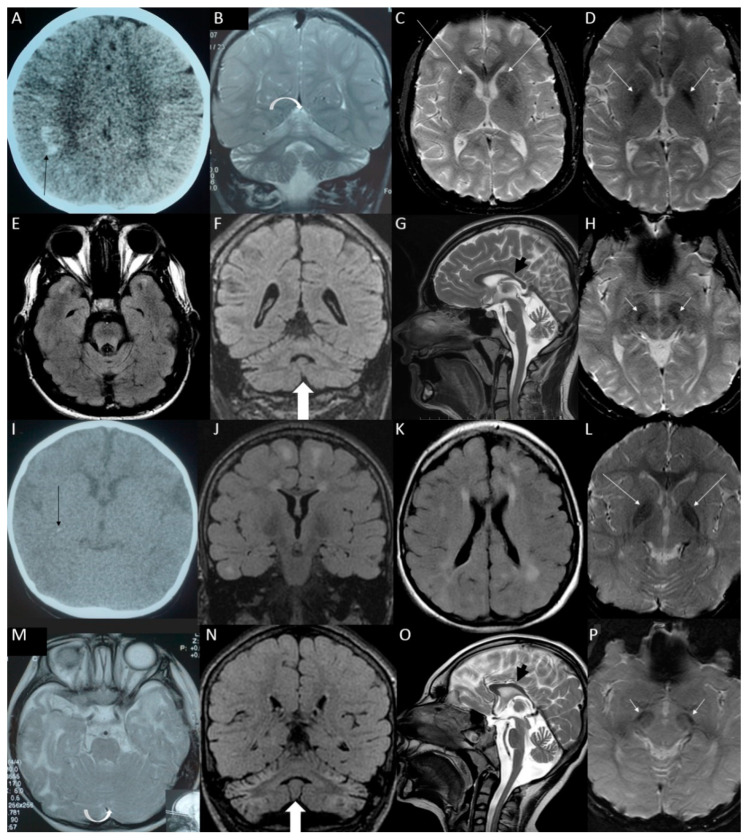
Brain imaging of sibling with *SUPV3L1* homozygous variant c.1093C>T:p.(Arg365Trp)—the older brother (patient S19a) in panels. (**A**) Computed tomography (CT) at the early childhood with marked subcortical calcifications in both frontal and parietal lobes (black arrow). (**B**) Subsequent magnetic resonance imaging (MRI) scans at the middle childhood with marked shrunken bright cerebellum (white arrow). (**C**–**H**) Brain MRI at the late adolescence. (**E**) FLAIR hyperintensities remained in the temporal poles only, (**F**) atrophic cerebellum but not so FLAIR-hyperintense as earlier and as in the younger sister (white arrow), (**G**) the corpus callosum is shorter and thinner than normal (black arrow), (**D**) iron or calcium deposits in the globi pallidi (white arrows), (**H**) in substantia nigra (white arrows) (**C**) and in the caudate nuclei (white arrows); brain imaging of sibling with *SUPV3L1* homozygous variant c.1093C>T:p.(Arg365Trp)—the younger sister (patient S19b): (**I**) CT at the early childhood revealed two punctate subcortical calcifications in the right cerebral hemisphere (black arrow). (**M**) MRI taken as the toddler with normal cerebellum with slight widening of the cerebellar sulci (white arrow). (**J**–**P**) MRI at the early adolescence: (**J**,**K**) myelination has progressed, but there are white matter hyperintensities on FLAIR sequence in both cerebral hemispheres, (**N**) shrunken bright cerebellum (white arrow), (**O**) shortened and thinned corpus callosum (black arrow), (**L**) iron or calcium deposits in the globi pallidi (white arrows) (**P**) and in substantia nigra (white arrows).

*SUPV3L1*: In a pair of siblings (S19a, S19b) with similar phenotype consisting of SM (−3.1 and −4.3 SD, respectively), delayed myelination, white matter abnormalities, profound DD, epilepsy and axial hypotonia, we identified a homozygous variant NM_003171.4: c.1093C>T: p.(Arg365Trp) inherited from healthy carrier parents. *SUPV3L1* gene encodes an RNA/DNA helicase, and the identified variant is reported in the gnomAD v3 population database with very low frequency (1.7 × 10^−5^) and no homozygous occurrences; 11/21 predictive algorithms classify it as damaging. Figure 1 contains detailed descriptions of brain imaging findings for both siblings.

*DHX9*: In patient T91 with SM (−2.8 SD), corpus callosum dysgenesis, DD, moderate ID, ataxia, hypotonia, convergent strabismus and dysmorphic facial features we identified a de novo variant NM_001357.5: c.3497G>C: p.(Arg1166Pro) in the *DHX9* gene encoding the DNA and RNA helicase. It is classified by 15/20 in silico predictions as damaging and is not reported in the gnomAD v3 database.

*MSI1*: In patient T20 with PM (−4.9 SD), mild ID, hypotonia, dysmorphic facial features and syndactyly trio analysis revealed the presence of two de novo variants, in *CBLC* and *MSI1* genes. One is a missense variant NM_012116.3: c.806G>T: p.(Arg269Leu) in the *CBLC* gene which encodes a member of Cbl family of E3 ubiquitin ligases. The variant found is not reported in the gnomAD v3 database and is classified by 16/21 predictive algorithms as damaging. The *MSI1* gene encodes a protein containing two conserved tandem RNA recognition motifs in which we identified a frameshift variant NM_002442.3: c.594dup: p.(Arg199GlufsTer180). It is not reported in the gnomAD v3 database, and although there is no record of this variant in the dbNSFP functional prediction database, it is classified as pathogenic by ACMG guidelines.

*ELFN1* and *CCDC112*: Using trio analysis we identified two de novo missense variants in the *ELFN1* and *CCDC112* genes in patient T62 with SM (−3.6), delayed myelination, asymmetric cerebral hemispheres, epileptic encephalopathy, hypotonia, sensorineural hearing loss, myopia and astigmatism. Variant NM_001128636.3: c.946C>T: p.(Arg316Cys) in *ELFN1* gene encoding postsynaptic protein that regulates circuit dynamics in the central nervous system is classified as damaging by 3/14 prediction algorithms and is not reported in the gnomAD v3 database. We also identified the variant NM_152549.2: c.59A>G: p.(His20Arg) in *CCDC112* gene encoding a coiled-coil domain containing protein. It is not reported in the gnomAD v3 population database and is classified as damaging by 1/20 in silico algorithms.

## 4. Discussion

**Figure 2:**
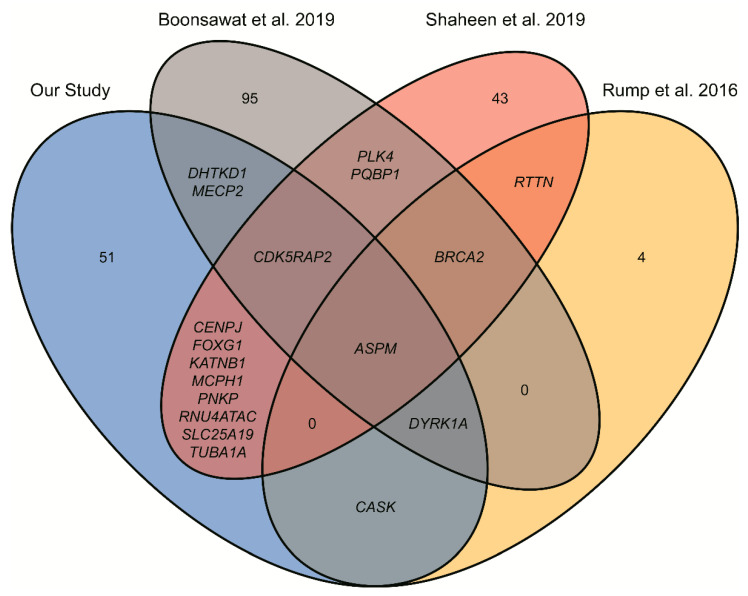
Venn diagram showing number and symbols of unique and shared genes identified in four studies on different microcephaly patients.

In our study we examined the full exomes of 191 Polish patients with clinically diagnosed microcephaly using routine exome sequencing technology. We identified 12 novel candidate disease genes and established a definitive molecular diagnosis in 19% of the patients, likely diagnosis for 18% of patients and found variants of unknown significance in genes associated with microcephaly in 6% of our patients. In aggregate, it gives a diagnostic yield of 43%, falling within the range reported for ES [7] and similar to results in previous studies regarding incidence of microcephaly [10,11] (29% and 48%, respectively). Comparison of these studies (Figure 2) reflects the high genetic heterogeneity of microcephaly, with the highest number of overlapping gene identifications at 8, between our study and that of Shaheen et al. (Figure 2). However, the only gene found in all four studies is *ASPM*, which could be explained by the fact that it is the most frequent gene mutated in patients with *MCPH* [8].

Patients with mutations in tubulin genes, the largest group with 15 cases in our cohort, demonstrated malformations of cortical development, with a wide range of disorders, including microcephaly, abnormal gyral patterns of cerebral cortex such as lissencephaly or polymicrogyria as well as presence of heterotopic grey matter. The high incidence of pathogenic or likely pathogenic variants in tubulin genes in our cohort underscores the relevance of microtubules and microtubule-associated proteins in brain development and the likelihood of microcephaly in the case of their disruption. However, this is the first study of microcephalic patients in which a high percentage of over 20% of cases result from mutations in tubulin genes.

Our study allows for the further delineation of phenotype of microcephalic patients, especially in the case of several genes in which mutations were identified in more than one patient. Our findings seem to confirm the existence of sex bias in the neurodevelopmental disorder with spastic diplegia and visual defects caused by variants in *CTNNB1*, as all our patients are females [33]. With recent reports of eight additional patients, five of whom were male, and four patients from our study the sex ratio in this disorder is approximately 63% females to 37% males [34,35]. In one case (S87) we identified a frameshift variant NM_001904.4: c.1665del: p.(Thr556HisfsTer14) that had been reported only once in the literature, while the other three (S42, S63, S145) were harboring more common pathogenic variants already reported in the ClinVar database. While all our patients with mutations in the *CTNNB1* gene had a similar phenotype with microcephaly (−2.3 to −4.3 SD), DD and abnormal muscle tone, patient S87 presented with uncommon finding of delayed myelination as evidenced on brain MRI studies. Three patients were found with strabismus, which supports the observation that ocular abnormalities are frequent findings in patients with *CTNNB1*-associated neurodevelopmental disorder [34].

Patients with congenital Rett syndrome due to pathogenic variants in *FOXG1* presented with typical clinical features such as progressive microcephaly, DD, corpus callosum anomalies, intellectual disability, epilepsy and strabismus but also rare findings including nystagmus (case S8 and S106) and previously unreported hypoplastic cranial nerves I and II (S8) [36]. 

Mandibulofacial dysostosis with microcephaly caused by heterozygous variants in *EFTUD2* gene is characterized by the presence of progressive microcephaly, dysmorphic features and hearing loss. Most of these symptoms were present in our patients (S39, S79, S80); however, patient S80 was found with a congenital heart defect that was not previously associated with *EFTUD2* defect. [37]. *PDHA1* mutations cause pyruvate dehydrogenase E1-α deficiency, a rare X-linked syndrome in which a high proportion of females with heterozygous variants manifest severe symptoms. Our patients (S37, S46, S89) with mutations in this gene were all females and displayed neurologic manifestations of this syndrome, with structural abnormalities in the central nervous system, mainly in the corpus callosum as well as DD [38]. Patients with pathogenic variants in the *PDHA1* gene usually develop microcephaly postnatally; however, in the case of patient S89, lower OFC was observed at birth, and the abnormalities of the brain were more severe when compared to the other two patients. This could be the result of the frameshift variant causing a premature stop codon that usually results in absent protein, whereas the other two patients had missense mutations, which are linked with less severe phenotype [39]. In contrast to those disorders with dominant inheritance pattern, bi-allelic mutations in *ASPM* cause autosomal recessive primary microcephaly 5, a rare syndrome belonging to the *MCPH* group. Our patients presented with OFC reduction without other congenital abnormalities, except for patient S49 with left side hemiplegia. Cortical malformations are quite a rare clinical finding in this condition; however, brain MRI revealed right brain hemisphere polymicrogyria in the previously mentioned patient and pachygyria in patient S52 [40]. All patients had compound heterozygous loss-of-function (LoF) mutations, as was expected considering the non-consanguineous cohort, and two patients (S49, S52) had a pathogenic variant NM_018136.5: c.7782_7783del: p.(Lys2595SerfsTer6) common in the European population [41].

Patients with variants in genes responsible for *MCPH* accounted for seven out of 71 resolved cases (9.9%). This frequency is almost two times higher than the prevalence (5.4% and 5.7%) reported in other cohort studies of microcephalic patients [10,11]. We demonstrated higher incidence of cases with mutations in *MCPH* genes which is surprising considering lack of reported consanguinity in our cohort, especially having taken into account the number of cases caused by autosomal recessive conditions, which constitute about a third (32.9%) of total resolved cases with about 70% of them caused by compound heterozygous variants. A similar number was reported in a study of 62 unrelated patients with microcephaly, where 37.5% of patients had syndrome with autosomal recessive inheritance pattern [11]. In contrast, a study with 35 patients of whom six were from consanguineous families reported this pattern of inheritance in 70% of cases [10]. We argue that in the event of no reported consanguinity or similarly affected siblings, dominant inheritance pattern should be more common in patients with microcephaly.

In our study we found four cases with more than one possible molecular cause that could lead to microcephaly, a phenomenon described as a dual molecular diagnosis and reported in approximately 5% cases with molecular diagnoses [42]. Table 2 presents the patients’ phenotypes and molecular findings compared with the most common clinical features reported in the literature found in patients with pathogenic variants in these genes. In each case, due to similar clinical features caused by mutations in distinct microcephaly genes, we could not exclude the possibility of overlapping phenotype. We identified a heterozygous variant in *TUBB2B* and a hemizygous variant in *HUWE1* in patient T50. Both genes cause syndromes with highly variable phenotype, especially in the case of *HUWE1*. Considering the X-linked inheritance pattern of this gene and the fact that this variant was inherited from a healthy mother, establishing a definitive molecular cause based solely on phenotype features is difficult. In a female patient S177 we identified a heterozygous de novo mutation in *HUWE1* and another de novo heterozygous variant in *SATB2* gene. In the case of previously mentioned *HUWE1*, the disruptions of which affect mainly males, recent studies report incidents of severely affected females, all of whom had de novo heterozygous variants [27]. On the other hand heterozygous variants in *SATB2* can lead to Glass syndrome, which is characterized by ID, dysmorphic facial features, cleft palate and crowded teeth, with variable clinical expression. Considering the phenotype of this patient, the de novo character of both mutations, as well as the high pathogenicity scores for *SATB2* variant, we cannot exclude the possibility of overlapping phenotype. In patient S188 we again identified two de novo heterozygous mutations in *GRIN1* and *KNCT1* genes. Disruption of these genes can lead to severe DD and seizures, both of which are present in this case. In the case of *GRIN1* both dominant and recessive inheritance pattern is reported, with causative heterozygous missense substitutions of de novo origin usually found in functional domains of the protein and resulting in LoF [43]. The variant c.1665G>T found in our patient is located between the ligand binding and transmembrane domains and was previously reported in a large Chinese cohort of patients, classified as likely pathogenic although without any information about patients’ clinical picture [44]. Considering similar phenotypes resulting from mutations of those genes we cannot denote a causal variant or rule out a possible dual molecular cause of phenotype present in our patient. Finally patient S78 with a homozygous variant in *MCPH1* inherited from both parents also had a pathogenic heterozygous variant in the *SCN8A* gene. Considering the phenotype of the patient, and likely benign reports regarding the variant identified in *MCPH1*, it is more probable that the mutation in *SCN8A* is the main cause of the disorder. However, without functional studies regarding the variant c.664T>C in *MPCH1* gene we cannot exclude a possible additive effect of this variant on the clinical picture of this patient.

This study identified novel variants in 12 genes, of which 11 were previously unrelated to microcephaly. The five high confidence candidate genes identified in our cohort are *SUPV3L1*, *DHX9*, *MSI1*, *ELFN1* and *CCDC112*. (Low confidence candidates and their possible link to microcephaly are included in Table S1). In siblings S19a and S19b we identified a homozygous variant in the *SUPV3L1* gene that encodes NTP-dependent RNA/DNA helicase, localized mainly in the mitochondrial matrix and in a smaller fraction in the nucleus [45,46]. In yeast, Suv3 homolog interacts with various proteins implicated in DNA replication, recombination and repair, as well as chromatin repair and genome stability [47]. In human cell lines *SUPV3L1* interacts with BLM and WRN proteins, involved in genome repair via homologous recombination [48]. Pathogenic variants in *BLM* cause Bloom syndrome, one of the clinical features of which is microcephaly [49]. Functional studies using knock-out of the *SUPV3L1* orthologue in mouse and zebrafish mutants showed embryonic lethality [50,51]; knock-down in mammalian cell lines results in apoptosis [46]. The amino acid substitution p.Arg365Trp localizes in the helicase C-terminal domain, which may impair function.

For patient T91, trio-analyses identified a heterozygous de novo variant in the *DHX9* gene. This encodes a helicase that unwinds both DNA and RNA and is important in fundamental processes such as DNA replication, transcriptional activation, post-transcriptional RNA regulation, mRNA translation and RNA-mediated gene silencing [52,53]. Loss of *DHX9* leads to an increase in circular-RNA-producing genes [54]. It also promotes formation of R-loops in cells with impaired splicing factors that can block DNA replication [55]. *DHX9* is also implicated in regulation of DNA transcription, translation, RNA processing and transport and maintenance of genome stability and is implicated in the development of many cancer types [56]. The initial data on *SUPV3L1* and *DHX9* may suggest implication of the aforementioned genes in normal brain development.

In two patients we found de novo variants in two distinct genes previously unassociated with microcephaly. In patient T20 we identified a heterozygous frameshift variant resulting in the introduction of a new stop codon downstream of the original stop in *MSI1* and a heterozygous amino acid substitution variant in the *CBLC* gene. Lack of expression in the brain and no obvious metabolic function of the latter gene raises questions regarding the possibility of it being a possible cause of microcephaly. The *MSI1* gene on the other hand encodes an RNA binding protein, which can inhibit translation initiation, stabilize RNA and influence alternative splicing [57,58,59]. Many mRNA targets of the mouse homolog Msi1 are involved in key cellular processes such as cell cycle, proliferation and survival [60]. The human protein *MSI1* binds to and is a translational activator of *MCPH1*, a gene associated with *MCPH* which has various role in the cell cycle. In particular, it acts as G2/M checkpoint arrest via maintenance of inhibitory phosphorylation of cyclin-dependent kinase 1 [61]. Additionally *MSI1* interacts with the Zika virus genome and enables viral replication [62]. Two siblings from a consanguineous family presenting with phenotype similar to MPCH had a homozygous missense variant in *MSI1*, as well as other homozygous variants in *ACACB*, *DKK4* and *DTX3L* genes [62]. Although our patient presents with a heterozygous variant, the unknown pattern of inheritance of the *MSI1* gene and resemblance of patient clinical features to *MCPH* were observed. It is therefore conceivable that heterozygous LoF variants in this gene cause microcephaly.

In patient T62 we found a de novo heterozygous variant in *CCDC112*, and another de novo heterozygous variant in *ELFN1*. *CCDC112* is a centriolar satellite protein and co-localizes with PCM1 which is a component of centriolar satellites and is essential for anchoring microtubules to the centrosome [63]. It is also implicated in ciliogenesis in male germ cells [64]. Defects in genes encoding centriolar satellite proteins, which are an important part of the centrosome, are linked with microcephaly [65]. In the case of *ELFN1* four heterozygous variants were reported in a cohort of Japanese patients with neurological disorders, such as epilepsy or autism spectrum disorder [66]. Functional studies show that *ELFN1* mutants display seizures, motor abnormalities and hyperactivity [67]. Moreover, the *ELFN1* protein is present in excitatory synapses, where it acts as a regulator of presynaptic release [68]. We speculate that the combined effect of variants in both of these genes may be responsible for the clinical picture of the patient (Supplementary Table S1). However, impact of disruptions of both the *CCDC112* and *ELFN1* genes is as yet largely unknown. Further information is therefore necessary.

Although the predictive algorithms and the current knowledge about the functions and interactions of proteins encoded by these genes indicate their potentially pathogenic nature, there is a need for functional studies or the identification of a larger number of patients with changes in these genes who display a similar phenotype to classify them as new genes, the mutations of which are responsible for microcephaly and other neurodevelopmental disorders.

## 5. Conclusions

Analysis of full exome data for 191 clinically diagnosed congenital microcephaly patients in our study provides a diagnostic rate of 37% and possibly pathogenic genetic variants in 12 genes previously unassociated with microcephaly. In addition, this is the first study of microcephalic patients in which such a large group of patients have mutations in tubulin-related genes. We also found that the number of patients with pathogenic mutations in *MCPH* genes is almost two times higher in comparison to similar previous studies. Our results indicate that in a group of patients with microcephaly and no reported consanguinity, the dominant inheritance pattern is more prevalent. Our observations expand the phenotypic and genetic landscape of microcephaly and support the routine use of exome sequencing when genetic etiology is suspected. Our findings further provide closer phenotypic delineation for variants in the most commonly disrupted genes in our own and other cohorts, facilitating differential clinical diagnoses for disorders associated with these genes.

## Figures and Tables

**Table 1:** Major clinical features and their frequency in patients enrolled in this study.

Clinical Feature	HPO Number	Frequency
Microcephaly	HP:0000252	191 (100%)
Primary microcephaly	HP:0011451	77 (40.3%)
Secondary microcephaly	HP:0005484	110 (57.6%)
Unknown onset	HP:0000252	4 (2.1%)
Cognitive impairment (DD/ID)	HP:0100543	168 (87.9%)
Abnormal cerebral morphology	HP:0002060	133 (69.6%)
Abnormal corpus callosum morphology	HP:0001273	55 (28.8%)
Abnormal myelination	HP:0012447	35 (18.3%)
Abnormal cortical gyration	HP:0002536	28 (14.7%)
Abnormal cerebellum morphology	HP:0001317	28 (14.7%)
Ventriculomegaly	HP:0002119	22 (11.5%)
Abnormality of the nervous system	HP:0000707	166 (86.9%)
Abnormal muscle tone	HP:0003808	99 (51.8%)
Seizure	HP:0001250	76 (39.8%)
Refractory status epilepticus	HP:0032867	25 (13.1%)
Epileptic encephalopathy	HP:0200134	10 (5.2%)
Hemiplegia/hemiparesis or tetraplegia/tetraparesis	HP:0004374 HP:0030182	22 (11.5%)
Abnormality of movement	HP:0100022	17 (8.9%)
Stereotypy	HP:0000733	13 (6.8%)
Short stature	HP:0004322	61 (31.9%)
Abnormal facial shape	HP:0001999	75 (39.3%)
Strabismus	HP:0000486	27 (14.1%)
Abnormal heart morphology	HP:0001627	19 (9.9%)
Hearing impairment	HP:0000365	13 (6.8%)

**Table 2:** Comparison of phenotype of patients with possible dual molecular diagnosis and most common clinical features of patients with mutations in those genes.

Patient	Sex	Phenotype	Variant and Inheritance	Zygosity	OMIM Syndrome	OMIM ID	Syndrome Main Features	Pubmed
T50	M	SM, ID, Dandy-Walker malformation, anterior commissure agenesis, dysmorphic facial features	*TUBB2B*-NM_178012.5 c.1171C>T p.(Arg391Cys) *dn*	het	Cortical dysplasia, complex, with other brain malformations 7	610031	DD, polymicrogyria, corpus callosum agenesis, brainstem hypoplasia	[26]
			*HUWE1*-NM_031407.7 c.11434G>A p.(Val3812Met) mat	hemi	Mental retardation, X-linked syndromic, Turner type	309590	DD, ID, hypotonia, speech delay, microcephaly, epilepsy, dysmorphic facial features	[27]
S78	M	PM, DD, axial hypotonia, epileptic encephalopathy, limb hypertonia, EEG abnormalities	*MCPH1*-NM_024596.5 c.664T>C p.(Cys222Arg)	hom	Microcephaly 1, primary, autosomal recessive	251200	ID, microcephaly, short stature	[28]
			*SCN8A*-NM_014191.4 c.5630A>G p.(Asn1877Ser) *dn*	het	Epileptic encephalopathy, early infantile, 13	614558	DD, epilepsy, myoclonus, extrapyramidal signs	[29]
S177	F	SM, DD, hypotonia, dysmorphic facial features	*HUWE1*-NM_031407.7 c.9208C>T p.(Arg3070Cys) *dn*	het	Mental retardation, X-linked syndromic, Turner type	309590	DD, ID, hypotonia, speech delay, microcephaly, epilepsy, dysmorphic facial features	[27]
			*SATB2*-NM_015265.4 c.490G>A p.(Asp164Asn) *dn*	het	Glass syndrome	612313	DD, speech delay, dental anomalies, behavioural difficulties, feeding issues, abnormal brain neuroimaging, dysmorphic facial features	[30]
S188	M	PM, DD, bilateral polymicrogyria, epilepsy, hypertonia, tetraplegia, nystagmus, strabismus convergent, cryptorchidism	*KCNT1*-NM_020822.3 c.1720G>A p.(Glu574Lys) *dn*	het	Epileptic encephalopathy, early infantile, 14	614959	DD, epilepsy, absent speech, hypotonia, spasticity, microcephaly	[31]
			*GRIN1*-NM_007327.4 c.1665G>T p.(Met555Ile) *dn*	het	Neurodevelopmental disorder with or without hyperkinetic movements and seizures, autosomal dominant	614254	DD, epilepsy, hypotonia, absent speech, movement disorders, spasticity, visual impairment, bilateral polymicrogyria	[32]

PM—primary microcephaly, SM—secondary microcephaly, DD—developmental delay, ID—intellectual disability, *dn*—de novo, mat—maternally inherited, het—heterozygous, hemi—hemizygous, hom—homozygous.

## Data Availability

The data presented in this study are available on request from the corresponding author. The data are not publicly available due to them containing information that could compromise research participant privacy.

## Supplementary Materials

The following are available online at https://www.mdpi.com/article/10.3390/genes12122014/s1, Table S1: Patients details, Table S2: Gene list, Table S3: Gene coverage, File S4: MultiQC report.
